# A Review on the Management of Hip and Knee Osteoarthritis

**DOI:** 10.1155/2013/845015

**Published:** 2013-09-28

**Authors:** Alexander MacDonald Wood, Timothy M. Brock, Kieran Heil, Rachel Holmes, Axel Weusten

**Affiliations:** ^1^Trauma and Orthopaedics, Wansbeck Hospital, Woodhorn Road, Ashington NE63 9JJ, UK; ^2^University of Glasgow Medical School, Glasgow, Scotland, UK

## Abstract

Arthritis is the most common chronic condition affecting patients over the age of 70. The prevalence of osteoarthritis increases with age, and with an aging population, the effect of this disease will represent an ever-increasing burden on health care. The knee is the most common joint affected in osteoarthritis, with up to 41% of limb arthritis being located in the knee, compared to 30% in hands and 19% in hips. We review the current concepts with regard to the disease process and risk factors for developing hip and knee osteoarthritis. We then explore the nonsurgical management of osteoarthritis as well as the operative management of hip and knee arthritis. We discuss the indications for surgical treatment of hip and knee arthritis, looking in particular at the controversies affecting young and obese patients in both hip and knee replacements. Patient and implant related outcomes along with survivorships are addressed as well as the experiences and controversies described in national joint registries.

## 1. Introduction

Osteoarthritis is the most common chronic condition affecting patients over the age of 70 [1]. It is estimated that in adults over the age of 30, up to 6% of adults are symptomatic of knee arthritis and around 3% are symptomatic of hip arthritis [2, 3]. The prevalence of osteoarthritis increases with age, and with an aging population [4], the effect of this disease will represent an ever-increasing burden on health care.

Osteoarthritis of the hip and knee is the most common cause of difficulty in walking [5]. It has a huge impact on the economy, with absence from work and early retirement exceeding 2% of the gross domestic product [6]. It is estimated that over 1 million total hip replacements are performed worldwide each year [7], and in the United States alone it is predicted that between 1995 and 2020 an additional 19 million people a year will be affected by arthritis [8].

We provide a comprehensive review of osteoarthritis in the hip and knee joints, including the disease process, risk factors, treatment options, and outcomes. This is based on a review of the literature using PubMed, Embase, and Medscape websites. Key searches used were hip and/or knee osteoarthritis. Searches were then substratified by pathophysiology, risk factors, treatment, and outcomes, using the most robust data available in each case.

## 2. The Disease Process

Osteoarthritis is an age related disorder, which can either be defined by the symptoms or pathology. It is characterised by damage to hyaline articular cartilage. It involves the whole joint and has subsequent changes to the subchondral surface involving bone remodelling. Patients may have a degree of synovitis associated with the arthritis as well as a generalised thickening of the capsule around the joint [9]. Radiographic changes are not a reflection of symptoms as many people with radiographic evidence of osteoarthritis will have no symptoms [10]. Radiographic changes include narrowing of the joint space, osteophytes, subchondral cysts, and evidence of overloading in the form of bony sclerosis. Osteoarthritis manifests as joint pain, although the cause of pain in osteoarthritis is unknown [11]. The disease also has time limited joint stiffness after inactivity.

The changes observed in osteoarthritis are thought to be a result of a complex network of biochemical factors [12]. Chondrocytes are believed to release proteolytic enzymes that break down the cartilage macromolecules and cause the changes to the cartilage seen in osteoarthritis. Cytokines involved in the process include interleukin 1 and TNF-alpha which are produced by activated synoviocytes, mononuclear cells, and the articular cartilage [12]. These inflammatory pathways also upregulated on top of the release of the cytokines by the cartilage [9].

At the cellular level this damage may be a result of an attempt by the chondrocytes to repair damaged cartilage. The cartilage will have an increased water content, with the collagen matrix gradually breaking down. There is also an associated decrease in the proteoglycan content of cartilage. These changes lead to an increase in proteoglycan synthesis combined with a large increase in proteoglycan breakdown. This leads to a relative increase in chondroitin sulphate in comparison to aging where there is a decrease in chondroitin. Likewise the keratin sulphate concentration moves in the opposite way to aging with a relative decrease in keratin sulphate.

The knee is the most common joint affected in osteoarthritis, with up to 41% of limb arthritis being located in the knee, compared to 30% in hands and 19% in hips [13].

## 3. Risk Factors for the Development of Lower-Limb Osteoarthritis

There is increasing evidence for the role of genetics in the development of osteoarthritis. Twin studies comparing the correlations of osteoarthritis at the knee show much greater occurrence amongst monozygotic than dizygotic twins, and it is thought that between 39 to 65 percent is attributable to genetic factors [14]. Other twin studies demonstrate a stronger genetic component to hand OA but weaker associations in the hip and knee [15]. In particular, the specific gene COL9A1 coding for type IX collagen has been highlighted as a susceptible locus for osteoarthritis in the hip in females [16], and the rs143383 polymorphism of the GDF5 gene consistently associated with the risk of knee osteoarthritis across different populations [17].

When the biomechanics of the hip and knee joint are altered or overused, the joint becomes more prone to the changes of osteoarthritis. Anatomical variants in hip morphology in the population have been linked to the development of osteoarthritis. In particular, there are strong correlations with the alpha angle, lateral centre edge angle, and extrusion index with the need for total hip arthroplasty [18]. Indeed these measurements could be used as an indicator to identify individuals who may go on to develop the disease later in life [18].

Obesity, previous knee injury, and sports activity have been shown to be risk factors for the incidence but not disease progression of OA of the knee [19]. In the Western world up to 23.6% of men and 23.8% of women are regarded as obese [20]. In varus knees, obesity affects incidence but not progression of OA in knees, and in knees with neutral or valgus alignment it affects the progression of OA [21]. Obesity is also a risk factor for hip osteoarthritis. Both men and women with a BMI > 28 have been found to be 1.7 times more likely to have hip OA than those with a BMI < 24.5 [2h].

Occupational habits are also known to be a risk factor, with the risk of knee OA significantly elevated in those whose job involves more than 30 minutes per day squatting, kneeling, or climbing stairs [22], with one study finding the prevalence rate of osteoarthritis in female cleaners over six times higher than expected [23].

The impact of sports activity and previous injury has been shown to be a risk factor for developing OA. In a group of retired England football players, 51% of players who retired through a football related injury were diagnosed with lower-limb OA compared with 25% of players who did not retire through injury [24]. Previous ACL injury in particular has been associated with almost 80% of sportsmen having radiographic OA at the knee fourteen years following injury [25]. Injuries to the hip from sports, falls, or road traffic accidents have been associated with an overall 4.3-fold increase in the risk of hip osteoarthritis [26].

In older adults, physical activity involving low muscle strength and high mechanical stress is a risk factor for developing knee OA, but there is not an increased risk with exercise intensity [27]. With an ageing population involved in sports and exercise, it is going to become even more important to be aware of the types of exercise that can be harmful. The number of people with osteoarthritis is going to increase, making the management of osteoarthritis of increasing importance.

## 4. Nonsurgical Management of Hip/Knee Osteoarthritis

The primary goals of the management of patients with OA are control pain and to bring improvement in function and health-related quality of life, with avoidance of toxic pharmacological effects [28]. The two approaches recommended for the medical management of hip or knee OA are nonpharmacological modalities and drug therapy [28].

Patient education, to include the patient's family or other caregivers, is an integral part of any OA treatment plan. National bodies such as Arthritis Care provide education materials in the form of pamphlets, newsletters, and online media ideal for educating OA patients. Patients should be encouraged to take part in self-management programs, as these have been shown to provide a decrease in joint pain and frequency of GP visits, increase in physical activity, and overall improvement in their quality of life [29]. Patients who receive personalised social support in the form of periodic visits or telephone calls have been shown to demonstrate a moderate to large improvement in pain and functional status without significant cost [30].

OA of the lower extremities may impair patients' ability to perform activities of daily living (ADLs) such as walking, bathing, dressing, using the lavatory, and household chores. Physical therapy and occupation therapy are central to the management of patients with functional limitations [28]. Physical therapists assess the joint and can provide instruction to the patient on how best to improve joint range and muscle strength as well as providing assistant devices such as canes and walkers. Occupation therapists can direct the patient to proper joint protection, the use of assistive devices, and improving joint function [28]. The 1995 ACR recommendations [29, 30] noted that the proper use of a cane is associated with a decrease in pain and improvement of function. 

Recent studies have also demonstrated the efficacy of an exercise program in improving muscle strength, mobility, and coordination and a decrease in the amount of paracetamol taken by patients with OA of the hip or knee [31]. The ability of elderly subjects with advanced OA to maintain conditioning levels may be problematic, as many are sedentary and deconditioned [32].

The American Academy of Orthopaedic Surgeons recommends that patients with knee or hip OA who are overweight, lose weight [33]. Few studies have examined this issue, but those who did have shown a significant improvement in knee OA after an appetite suppressant and exercise program [34]. 

Pharmacological treatments should be used in addition to nonpharmacological measures, as drug therapy for pain management is most effective when combined with these measures [35]. The majority of medications are focused on symptom management. For many patients treatment the relief of mild-moderate joint pain from a simple analgesic (paracetamol) is comparable with that achievable with nonsteroidal anti-inflammatory drugs [33, 36–40].

In more severe joint pain, studies have shown that patients have a greater preference for NSAIDs (diclofenac) than for paracetamol, because of its superior improvement in pain relief and function, although many patients continue to take paracetamol [41–44].

Cyclooxygenase 2 specific inhibitors (celecoxib and rofecoxib) have been shown to be more effective than placebo and comparable in efficacy with both ibuprofen and diclofenac in patients with hip and knee OA [45–49]; however, like NSAIDs, COX-2-specific inhibitors can cause renal toxicity. 

NSAIDs may be used with gastroprotective agents such as proton pump inhibitors (famotidine and omeprazole) both of which have been effective in NSAID gastropathy [50–53]. Prostaglandin E1 analogs (misoprostol) have also been found to be effective [54]. H2 blockers have not been found to be as effective [52].

In individuals with OA of the knee suffering mild-moderate pain, who do not respond to paracetamol and do not wish to take systemic therapy, the use of topical analgesics (methyl salicylate or capsaicin cream) is appropriate.

An alternative approach to the use of oral agents to relieve joint pain is intra-articular therapy with hyaluronan or glucocorticoids. Trials with hyaluronan preparations have found pain relief comparable with that of NSAIDs [55–57], though they are not approved for the treatment of hip OA.

Centrally acting oral analgesic (tramadol) is approved for moderate to severe pain relief; though few studies have been carried out, its efficacy has been found to be comparable with Ibuprofen for patients with hip and knee OA [58]. Patients who do not respond to or cannot tolerate tramadol may be considered for more potent opioid therapy [59].

Research into glucosamine and chondroitin sulphate has shown a reduction in the deterioration of cartilage by stimulating the synthesis of proteoglycans and inhibiting the proteolytic enzymes; however, in the largest randomised placebo-controlled study there was no difference in pain and function than with the placebo [60].

There is a degree of controversy with regard to intra-articular corticosteroid injections for osteoarthritis. The current literature has demonstrated that corticosteroids in osteoarthritis of the knee shows a statistically and clinically significant reduction in pain when compared to placebos, and that triamcinolone is more effective than other corticosteroids [61, 62]; however, other papers and reviews have concluded that whilst there is a reduction in pain, this effect is likely to be limited for 3-4 weeks but unlikely to continue beyond that [63]. The literature from the British Institute for Health and Clinical Excellence advise that corticosteroid injections should be considered as an adjunct to core treatment for the relief of moderate to severe pain in people with osteoarthritis [64]. The Academy of American Orthopaedic Surgeons feels that given the conflicting literature, this cannot be recommended given inconclusive evidence [33].

Disease modifying drugs aim to treat osteoarthritis at an earlier stage. These include cell based products, including suspensions, delivery vehicles, scaffolds, and more advanced tissue engineering. Whilst promising, the heterogeneity and difficulty in early diagnosis of osteoarthritis have hampered this so far, and large, multicentre clinical studies are required.

## 5. Total Knee Replacements

Total knee replacement is indicated in patients with severe osteoarthritis suffering extensive pain and deformity, where conservative measures have failed. 

Contraindications include active infection, critical limb ischaemia, and a nonfunctioning extensor mechanism.

The decision to operate on the obese patient is based on the benefits of a reduction in pain and increased function, versus the risk of perioperative morbidity and mortality. These are well documented and include infections, respiratory complications, and thromboembolic disease. 

There is conflicting literature with regards to outcome in obese patients following total knee replacement. Despite this, as mentioned previously, there is a consistent recommendation that it is beneficial for obese patients to lose weight.

Some studies suggest that obese patients have lower patient reported outcomes and quality of life [65]. Foran et al. evaluated seventy-eight total knee replacements in obese patients and compared them with matched, nonobese controls. They found that obese patients had significantly lower outcome scores, measured by the knee society objective and functional scores. They also had a significantly higher rate of revision compared to the nonobese group [66]. This contradicts other work, which has shown no difference in outcome between the groups [67, 68].

Singh et al. [69] found no difference in pain outcomes in obese patients at two- and five-year followup. A large, recent study assessing the British National Joint Registry assessed patient-reported outcome measures in 13,673 patients undergoing total knee replacement [70]. The authors found that there was no different in patient reported outcome measures in obese patients, although wound complications were significantly higher.

Despite the conflicting literature, patient satisfaction is generally improved in obese patients following total knee replacement [71, 72], and therefore, it should be utilised in severe disease after discussing the relative benefits and risks of surgery.

Whilst total knee replacement is well established in the elderly with predictable and reproducible results [73–75], many orthopaedic surgeons are reluctant to perform TKA in younger patients which may be a result of concerns that high levels of activity may lead to increasing wear and aseptic loosening. This is also compounded with the increased technical challenges, and complications associated with multiple revisions are additional causes for concern in these patients [76].

There are a few reports showing favourable outcomes in the under-55 age group [77, 78]. Whilst most of these series contain a majority of patients suffering from rheumatoid arthritis and performed by surgeons in specialist centres [79], there are some more specific papers that demonstrate that cemented total knee replacement in patients under the age of 55 has a 98.2% survivorship at ten years [80]. Some specialist centres have reported survivorship between 87 to 99% in young patients undergoing a cemented total knee replacement at a medium-term follow up between 6 to 14 years [80–82]. In a case matched series it was also suggested that younger patients achieve similar results from TKA, in terms of pain and function, which are comparable and even superior to older patients when matched for ASA, diagnosis, and BMI [83]. Therefore, a younger age should not preclude patients from having a total knee replacement although in cemented components there still remains general anxiety about the osteolysis and loosening as a result of wear debris from the perceived multiple cyclical loading.

When advising people about total knee replacements, it is important to be able to quantify likely outcomes. The national joint registries provide one source of information, which are discussed later. There are a number of papers in the literature looking at the survivorship of the total knee replacements with recent papers on commonly used implants showing that total knee replacements can have a 98% survival at 10 years for revision for aseptic failure and 95.9% survival at 10 years for revision for all causes [84]. These results demonstrate that total knee replacements can last a number of years without revision; however, unless a centre is part of a registered trial it is important to use implants with a proven track record to ensure that patients can be fully consented about the risk of revision.

There is also an increasing trend to start reporting patient related outcome when reporting the success of total knee replacements. It is possible to assess the outcomes using a number of methods; one of these is the American Knee Society (AKS) scoring system described by Insall et al., 1989 [85]. The American Knee Society scoring system is separated into a Knee score and the Function Score to prevent confounding of the Knee Scores by increasing infirmity. Whilst generally there are significant improvements in the AKS across all joint types, the results can be variable with Zaki et al., 2007 [86], and Hunter et al., 2009 [87], reporting results which were not as favourable as studies like the Wrightington and Dalury's studies [86, 88] which both showed a significant improvement and a mean postoperative score of 50.

Other reporting methods include the Oxford Knee Score and this score, often demonstrates that patients will get continued improvements up to one year postoperatively [84]. It is also possible to use other scores like the SF-12 [89] to measure health status; these results are important in the early postoperative period as early results have been shown to be predictive of overall satisfaction rate by Scott et al., 2010 [90].

## 6. Total Hip Replacements

Osteoarthritis remains the most common indication for hip arthroplasty. The most important factors that hip replacement must address are pain relief, the ability to return to work, and an increase in the patients level of activity with particularly emphasis on walking capacity. It is imperative that patients are satisfied with the replacement, and examination parameters should be improved [91]. This has changed from the early indications for total hip replacements which was in the elderly patient crippled with arthritis, and increasing young patients are presenting with arthritis, wanting to improve their quality of life as well as wanting to continue physically demanding activities [92].

Rest pain and pain with activity as well as functional limitations are agreed upon as indications for surgery. In particular, intractable pain at night, significant reduction in walking distance, and inability to put shoes or socks on or cut your own toenails without help are classic complaints preoperatively. X-ray findings alone do not suffice as an indication for a joint replacement [93].

The most common sites for the pain are the groin and anterior and lateral thigh, the buttock, and the knee [7]. The examination of the arthritic hip may show an antalgic or trendelenburg gait, although this sign is unreliable [94]. It is normally possible to demonstrate pain on flexion and internal rotation, along with a decreased range of motion, along with a decrease in abduction and adduction of the hip and hip flexion contractures, and pain at extremes of motion. Leg length discrepancy greater than 1.5 cm, both true and apparent, can also be reliably detected [94].

Hip arthritis commonly affects the ability to climb stairs and causes difficulty in walking [5]; this loss of function along with difficulty in putting on shoes and socks and other activities of daily living are also considered indications for total hip arthroplasty [93, 95], whilst the presence of sepsis is an absolute contraindication for surgery [96].

Obesity is associated with an increased risk of osteoarthritis of the hip due to the pressure the extra weight exerts on the joint [97–99]. Despite this around 81% of European orthopaedic surgeons consider obesity to be a poor prognostic factor in patients undergoing elective THR [100].

 Obesity is thought to increase operative time in total hip replacement [101], thromboembolism [102, 103], blood loss [104], postoperative infection [105, 106], and dislocation rates in the short term [107, 108].

In the United Kingdom the national guidelines state that patient specific factors, including obesity, should not be barriers for referral for joint replacement surgery [109].

In a recent prospective study, obesity was shown to have a negative impact on the five-year outcomes following THR; however, it found that the overall benefit, whilst diminished in obese patients, is still positive [64]. In countries which regularly perform bariatric surgery, a recent study found that obese patients who have bariatric surgery prior to having hip arthroplasty performed better than those who had hip arthroplasty first [110].

Younger candidates for total hip replacement provide a more complex management decision; they have higher functional demands and require a longer implant life expectancy. Despite this, long-term studies of young patients undergoing total hip replacement with cement have demonstrated inferior durability compared to older patients [111].

Takenaga et al. investigated cementless total hip replacements in 100 patients under 50 years old at a minimum of ten-year followup against an age-matched group undergoing cemented total hip replacement [112]. The authors found that aseptic and radiographic loosening was lower in the cementless group; however, revision for polyethylene wear resulted in similar total reoperation rates in the long term.

Contrary to this, a large study by Hailer et al. investigated Swedish Joint Registry data investigated 170,413 patients who underwent total hip replacement [113]. They found inferior survival of uncemented total hip replacements at ten years primarily due to the poor performance of uncemented acetabular cups, and this was the same for the younger cohorts.

Total hip replacement prostheses are wide-ranging. In the UK, over 100 femoral and acetabular components are currently marketed. They are generally divided into cemented, cementless, and hybrid designs. Randomised controlled trials have attempted to compare different prosthesis types; however, studies are often flawed, by small numbers or inadequate followup.

A systematic review and meta-analysis by Morshed et al. compared cemented and cementless prostheses [114]. The authors concluded that there was no difference between groups in revision rate. However, cemented prostheses were shown to have superior survival rates compared to cementless prostheses in all age groups.

Pennington et al. studied quality of life and costeffectiveness after hip arthroplasty in 30 203 patients in the United Kingdom [115]. The authors found that whilst the cemented prosthesis was the cheapest, the hybrid prosthesis was associated with the highest quality of life, as measured by the Oxford knee score and EQ-5D-3L satisfaction scores, and was the most cost effective. The authors felt that cementless prostheses did not improve health outcomes sufficiently to justify their high cost.

McMinn et al. investigated revision rate and mortality rate using the National Joint Registry in the United Kingdom [116]. The study investigated approximately 250 000 cases. Due to large baseline differences in patient characteristics, unadjusted comparisons were not reliable or appropriate. The authors found that mortality rates were higher in cemented procedures compared to cementless ones (0.4% versus 0.2% at 30 days, 0.8% versus 0.5% at 90 days, and 21.8% versus 12.3% at eight years). Implant survival at six years was 98% in cemented procedures and 97% in cementless ones. The authors conclude that although differing patient characteristics may have confounded results, there was a small but significant increase in revision rate in cementless procedures and a small but significant increase in death in cemented procedures.

## 7. Joint Registry Data

The Swedish Knee Register was founded in 1976 and is regarded as a trailblazer in evidence-based surgery [117]. The Swedish Hip Register started in 1979 [118], and soon Norway and Denmark followed ahead of Europe, Canada, and Australia [118]. These large national databases overcome single institution bias and allow independent and robust data in a heavily industry driven market. National registers can highlight changes in trends, such as the use of certain surgical approaches, techniques (cemented versus uncemented), common complications, or catastrophic failure of an implant [119].

Altogether, the Nordic Arthroplasty Register Association reviewed 280,201 total hip replacements in a recent study in 3 countries over one decade [120]. Osteoarthritis accounted for the majority of cases, whereas childhood diseases for 3.1%, 1.8%, and 8.7% in Denmark, Sweden, and Norway, respectively. Females accounted for 60% of the patients in Denmark and Sweden and 70% in Norway. Cemented THRs were used in 46% of patients in Denmark, in 89% of patients in Sweden, and in 79% of patients in Norway. Their 10-year survival ranged from 92% in Denmark to 94% in Sweden and 93% in Norway [120].

Cemented prostheses showed significantly better overall survival results than uncemented/hybrid implants in all three countries. Despite these findings, orthopaedic surgeons in Denmark followed the trend of many continental European countries and the USA towards using more uncemented prostheses despite these findings. 

In the UK, the ratio between cemented and uncemented implants appears unchanged, albeit with an increase in hybrid procedures. The National Joint Registry for England and Wales reports 71,672 primary hip replacements undertaken in 2011, 36% were cemented, 41% cementless [121].

Knee arthroplasty, on the other hand, is the most common surgical treatment of osteoarthritis worldwide. In 2011 it accounted for 79,516 primary knee replacements in the UK alone. Of these, 91% were total knee replacements [121]. A comparison of 25,004 primary TKRs on the Norwegian knee arthroplasty register with 56,208 from the Kaiser health care system in the USA showed a seven-year survival of 94.8% and 96.3% respectively [122, 123]. The Swedish joint register states that the 10-year risk of revision after primary TKA was 4%.

In elderly patients over 70 years of age, this risk may even be lower. In the young patient cohort, however, treatment options and patient satisfaction are less advantageous [122]. Minimally invasive surgery (MIS) and unicondylar knee replacements were intended to deal with this problem. NJR data in the UK shows an increase in the use of MIS in unicondylar knee replacements from 37% in 2004 to 48% in 2011.

The use of a smaller surgical incision and therefore less soft tissue trauma has been advocated to facilitate a faster surgical rehabilitation. Though the evidence to support this has been controversial [124].

NJR data in the UK shows an increase in the use of MIS in unicondylar knee replacements from 37% in 2004 to 48% in 2011. Indications for a partial (unicompartmental) knee replacement include isolated OA to only one compartment, no fixed varus deformity over 10 degrees or fixed valgus deformity over 5 degrees. Contraindications include inflammatory arthropathy and ACL deficiency as well as previous meniscectomy in the contralateral compartment. Hence why only 6% of patients meet these criteria [125].

However, patient reported outcome measures (PROMs) do not seem to support the increased use of unilateral knee replacements [124]. Baker et al. examined linked national joint registry (NJR) data comparing 23,393 TKRs and 505 UKRs after adjustment for case-mix differences using multiple regression analysis. Their joint registry results showed no difference between TKR and unicondylar knee replacement with regards to knee-specific scores (EuroQol, EQ-5D, Oxford Knee Score) or general health outcomes. The authors therefore questioned the use of unicondylar implants in the presence of significantly higher revision rates in worldwide registries.

Similarly, the use of computer navigated arthroplasty instead of surgeons using simple templates and jigs failed to show any improvement in the literature [126]. A new development in arthroplasty is the use of patient specific TJR where components are produced to precisely match a preoperative MRI scan of the patient's joint. However, there are insufficient numbers of independently funded procedures so far to assess any significant difference in outcome in a heavily industry driven market.

In summary, joint replacement surgery for osteoarthritis leads to high patient satisfaction and longevity. However, further monitoring on national registries is needed to determine the best implant choices and to further optimise current treatment options.

## 8. Conclusion

Osteoarthritis is the most common chronic condition affecting patients over the age of 70, and the knee joint is the most common joint affected. We have discussed the current concepts in osteoarthritis in the hip and knee, to provide the reader with increased understanding about the causes, treatment options, and likely outcomes in different patient groups as well as some of the ongoing controversies surrounding different types of implants.
